# Long-Term Artificial Selection for Increased Larval Body Weight of *Hermetia illucens* in Industrial Settings

**DOI:** 10.3389/fgene.2022.865490

**Published:** 2022-06-15

**Authors:** Elena Facchini, Kriti Shrestha, Estelle van den Boer, Petra Junes, Gaya Sader, Katrijn Peeters, Eric Schmitt

**Affiliations:** ^1^ Hendrix Genetics Research Technology & Services B.V., Boxmeer, Netherlands; ^2^ Protix Biosystems B.V., Dongen, Netherlands

**Keywords:** black soldier fly, artificial selection, genetic improvement, body weight, insect farming industry

## Abstract

Black soldier fly (*Hermetia illucens*) farming has exponentially increased in recent years due to the ability of its larvae to efficiently convert low-grade organic materials into high-value food, feed, and technical products. There is a need to further improve the efficiency of production, to meet the rising demands for proteins in the feed and food industries under limited resources. One means of improvement is artificial selection, which has been widely applied in plants and in other livestock species. In 2019, a genetic improvement program was started with the aim to increase larval body weight in black soldier fly larvae. In this paper, we present the outcomes of this breeding program after 10, 13, and 16 generations of selection. The performance of the selected body weight line was compared to the base population line over six experimental rounds under different environmental conditions. Under automated production settings, an average increase of +39% in larval weight, +34% in wet crate yield, +26% in dry matter crate yield, +32% in crude protein per crate, and +21% crude fat per crate was achieved in the selected line compared to the base population line. This research demonstrates the potential contribution of artificial selection to improve efficiency when farming black soldier flies in industrial settings. Further research is needed to fully unlock that potential.

## Introduction

**FIGURE 1:**
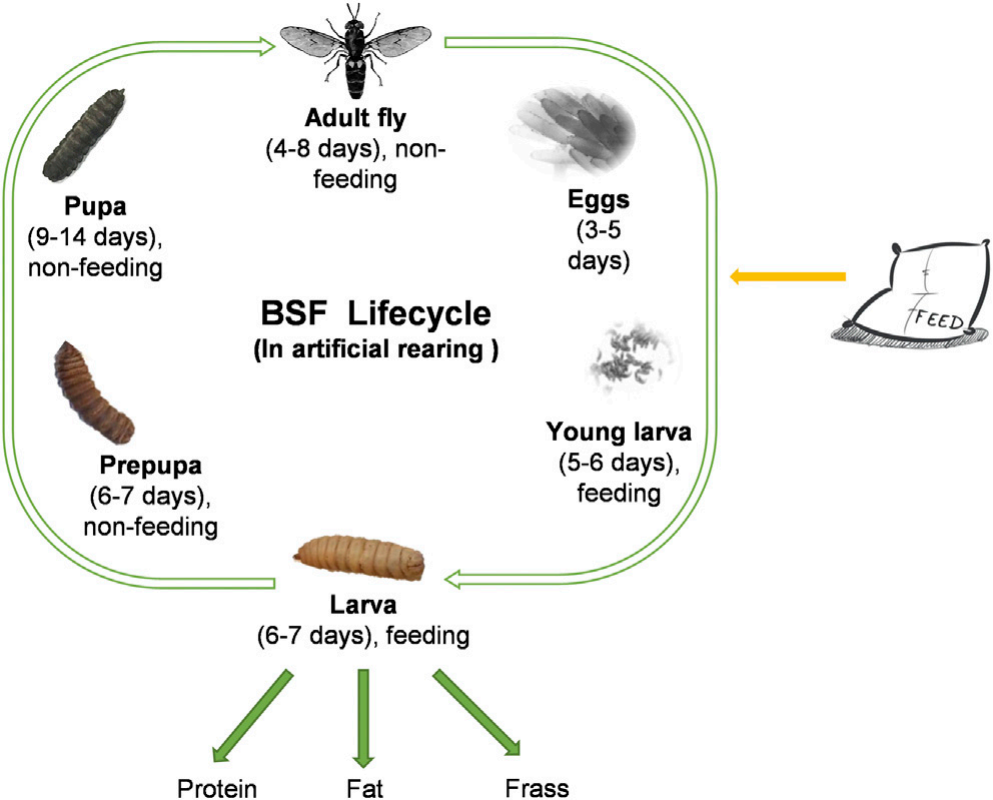
Life cycle of *Hermetia illucens*.

The black soldier fly (BSF), *Hermetia illucens* L. (Diptera: Stratiomyidae) is a detritivorous insect native to the Neotropical region (Brammer and Von Dohlen, 2007). It is now cosmopolitan in distribution, owing to its widespread utilization for organic waste remediation that yields various products of human interest (Li et al., 2015; Nguyen et al., 2015; Oonincx et al., 2015; Parra Paz et al., 2015; Leong et al., 2016). The BSF life cycle lasts 40–45 days under artificial mass rearing depending on factors like temperature (Tomberlin et al., 2009; Holmes et al., 2016; Chia et al., 2018; Opare et al., 2022) and diets (Gobbi et al., 2013; Nguyen et al., 2013; Zhou et al., 2013; Myers et al., 2008; Miranda et al., 2019; Singh et al., 2022). It consists of five distinct life stages: egg, larva, prepupa, pupa, and adult fly (Figure 1). The BSF life cycle starts with eggs, which hatch into young larvae. The larval stage is the only stage when the animal feeds. Once larval stage is over, the larvae transform into pre-pupae. The pre-pupae will become pupae, and at the end of metamorphosis the flies hatch from the pupal case. An industrial production cycle for BSF typically consists of four production stages: the breed (flies that mate to yield eggs), the nursery (eggs hatching into young larvae), the rearing (young larvae to harvestable larvae), and the pupation (prepupae to pupae that will eclose to adult flies). Usually, the breed lasts 7–13 days, the nursery lasts 5–6 days, the rearing lasts 6–7 days and the pupation lasts 15–21 days. The length of each stage is highly influenced by environmental conditions (temperature, humidity, and feed). The key production stage is the rearing, where young larvae voraciously convert organic waste into value derivable products such as protein meal, fat, and frass.

In the last 10 years, the commercial mass rearing of BSF and other insects for food and feed has grown rapidly. Interest in the species stems from the ability of the larvae to convert low-grade organic materials into high-value food, feed, and technical products. The insect farming industry for animal feed and pet food is a growing industry, with investments exceeding €1 billion since its establishment and the potential to generate 25,000 jobs by 2030 in Europe alone (IPIFF, 2021). The recent approval of insect-derived processed animal proteins (PAPs) by the European Commission for use in monogastric livestock diets, in addition to aquaculture and pet food diets, is expected to further boost the insect farming industry [European Commission, 2021 Commission Regulation (EU) 2021/1372 amending Annex IV to Regulation (EC) No 999/2001]. There is potential for insect-derived PAPs to replace soymeal and fishmeal as a more sustainable source of protein in animal feed (Smetana et al., 2019; Gasco et al., 2020). In addition, they are reported to benefit animal health and welfare (Ipema A. F. et al., 2020; Ipema AF. et al., 2020; Star et al., 2020; Mouithys-Mickalad et al., 2021). At present, the demand for insect-derived PAPs is higher than the supply, and the demand is expected to grow 50 times by 2030, globally (Rabobank, 2021). The pet food sector is currently the largest user of insect-derived PAPs, while the aquaculture feed sector is potentially the largest addressable market (Rabobank, 2021). However, the lack of supply volume remains one of the key constraints for the use of insect-derived PAPs in aquaculture. Meanwhile, the pet food sector could absorb higher volumes if available (Rabobank, 2021). The insect protein sector should reply to such growing demand by expanding production plants, while simultaneously improving the production systems. Despite BSF’s inherent efficiency (van Huis, 2016), further improvements would have a significant impact from an economic and environmental point of view (Smetana et al., 2019). One means of improvement is artificial selection, which has been widely applied in plants and in other animal species, but not in BSF. Artificial selection in livestock relies on selecting and mating animals with preferred genotypes, to move the population mean to the desired direction. Genetic improvement in livestock is directed towards traits such as faster growth, higher finishing weight, and improved feed conversion ratios. Acknowledged examples of artificial selection can be found in poultry, pigs, and dairy cattle. For example, in broiler chicken, improvements of 43% in feed conversion ratio (FCR) and 243% in body weight were achieved in 45 years of artificial selection (Hume et al., 2011). The environmental impact caused by Green House Gasses (GHG) emissions from broiler production decreased by 1.6% per year from 2014 to 2018 (De Haas et al., 2021). Similarly, in pigs, improvements of 27% in FCR and 37% in lean meat yield were achieved in 45 years of artificial selection (Hume et al., 2011), while GHG emissions from pig production decreased by 0.6–0.7% per year from 2014 to 2016 (De Haas et al., 2021). In dairy cattle, milk production increased by 67% in 45 years of artificial selection (Hume et al., 2011). In honeybees, artificial selection over a period of 10 years resulted in genetic improvement of 17% for honey yield, 15% for gentleness and 15% for the Varroa parasite resistance index (Hoppe et al., 2020). In mealworms, pupal body weight of *Tenebrio molitor* increased 66% after 8 years of artificial selection (Morales-Ramos et al., 2019). In addition, various studies reported body mass to be a medium to highly heritable trait in different insects’ orders, with heritability estimates ranging from 0.2 to 0.6 (Dingle et al., 1980; Iyengar and Eisner, 1999; Saastamoinen, 2008; Jumbo-Lucioni et al., 2010; Ellen et al., 2016; Facchini et al., 2021). These heritability estimates indicate that the population mean for body mass can be increased through artificial selection.

No reports are available on the impact of artificial selection in BSF. Setting up a successful breeding program in BSF, that would achieve comparable results as in livestock or other farmed insects, would be a considerable scientific accomplishment.

In 2019, a genetic improvement program was started with the aim to increase larval body weight in black soldier fly larvae. In this paper, we present the outcomes of this breeding program after 10, 13 and 16 generations of artificial selection. The aim of this study was to determine the differences in the larval performance of the Body Weight (BW) line compared to the Base Population (BP) line over multiple successive rounds, including multiple industrial production conditions. Our research findings are relevant as they provide an indication of the role that artificial selection may play in improving the performance of BSF and other insect species, as well as the direction that this practice may set for the economic and environmental implications of their cultivation.

## Materials and Methods

### Black Soldier Fly Lines

Two lines of *H. illucens* larvae were compared in the study: the BW line (*H. illucens,* selected for higher Body weight) and the BP line (*H. illucens,* the in-house strain, not selected for higher Body weight). The BW line was split off from the BP line in March 2019 and kept in the Genetics lab (Bergen op Zoom, the Netherlands), where the breeding program was commenced. Each generation, the BW line was selected for increased larval body weight, while minimizing the increase in inbreeding. Both the BW line and the BP line were maintained completely indoors, under controlled environmental conditions.

### Experimental Setup

To compare the BW line with the BP line, six successive rounds of experiments were carried out throughout the period from May 2020 to June 2021. Each round was characterized by a different setup, aimed at representing different production settings. Table 1 summarizes the setups which differed in feed type, rearing environment, larval density, and feed ration. The feed rations across rounds were defined by operators depending on the animals’ needs (except for round two). In other words, the feed ration was not a pre-determined setup we chose to vary, but a variable that changed (and was measured) according to other factors within a setup. For example, when different densities were used, different feed rations were given, as the feeding amounts are highly dependent on the larval density and available volume in a crate. The pattern code indicates the round-line-density, where larval density is coded in ascending order from D1 to D6.

**TABLE 1:** Summary of the experimental setup.

Round	Feed type	Environment	Line	Pattern code^a^	Δ Density^b^	Δ Feed ration^b^
1-May-20	A	1	BW	1-BW-D4	−15%	21%
			BP	1-BP-D4	−15%	4%
2-Sep-20	A	2	BW	2-BW-D1	−55%	135%
			BP	2-BP-D1	−55%	135%
3-Jan-21	B	2	BW	3-BW-D2	−27%	58%
			BP	3-BP-D2	−27%	12%
4-Mar-21	B	2	BW	4-BW-D3	−18%	49%
			BW	4-BW-D5	0%	30%
			BW	4-BW-D6	18%	10%
		3	BP	4-BP-D5*	0%	-2%
5-May-21	B	3	BW	5-BW-D5	0%	30%
			BP	5-BP-D5*	0%	0%
6-Jun-21	B	3	BW	6-BW-D5	0%	22%
			BP	6-BP-D5*	0%	0%

a Pattern code indicates round-line-density, where density is coded in ascending order from D1 to D6.

b Density and feed ration (grams of feed per larva) are represented as deviations (Δ) in percentage from a common benchmark set at industrial production conditions for the Base population line (BP).

* Indicates the BP, comparison groups that were extracted from production batches.

The first round took place at the Protix pilot facility in Dongen (Netherlands; Environment 1). Six crates per line were set up in parallel. The same rearing conditions were applied to all crates. Larval density and feed type (feed A) were used according to the production protocol developed at the Protix facility in Dongen.

The second round aimed at reconfirming the findings from the first round at the Protix facility in Bergen op Zoom (Netherlands; Environment 2). Six crates per line were set up in parallel. Animals were reared under the same conditions in a semi-automated system and with a lower larval density compared to round one, on feed type A.

In the third round, six crates per line were set up in parallel in Environment 2. Compared to the first two rounds, an in-between larval density was tested, and the animals were fed standard production feed B.

The fourth round sought optimal conditions for the BW line in a fully automated industrial setting. Three different larval densities for the BW line were tested in Environment 2. Six crates with the BW line were reared for each density group, and the animals were fed feed type B.

The fifth and the sixth rounds aimed at testing the BW line’s performance in a fully automated production system at the Protix facility in Bergen op Zoom (Environment 3). The data collected for the BW line in rounds five and six were taken from eight and ten crates from the automated production line, respectively.

For the first three rounds, a control BP group was set up in parallel next to the BW line. Operators were blinded to avoid bias in treating the animals differently based on the line. In round two, operators were instructed to deliberately feed the same amount on crate level, even if the animals indicated different needs. In round one and three, operators were allowed to feed different amounts on crate level, according to the operator’s discretion. As the experimental set up in rounds one and three were blinded, yet different feed rations were given to both lines, this reflects that the BW and the BP lines have different feeding needs. To confirm the different feeding needs of the two lines, a separate feeding trial was carried out at the Genetics Lab in Bergen op Zoom. Two feeding regimes were tested: Standard (ST) and *ad-libitum* (AL) feeding rations. For the standard feeding ration, the BW and the BP lines were fed according to their needs, i.e., 1.82 and 1.67 g of feed per larva, respectively. Meanwhile, for the *ad-libitum* treatment, both lines were fed abundantly, i.e., 2.5 g of feed per larva.

For the last three rounds, no control BP group was setup in parallel next to the BW line. Therefore, from the fourth round onwards, data from randomly selected crates from the BP parallel production batches, 15 flanking days before and after the BW experimental batch, were used for comparison (145 BP crates in round four, 35 BP crates in round five, 70 BP crates in round six). The feeding amounts for the BW line henceforth were determined based on the experience from previous three rounds, to maximize the output per crate within the existing production system.

Three different generations of the BW line were used in this study. For the first, second, and third round, the BW animals were sourced from the Genetics lab in May 2020, September 2020, and January 2021, respectively. The BW animals sourced in May 2020 (first round) were selected for 10 generations; the BW animals sourced in September 2020 (second round) were selected for 13 generations, and the BW animals sourced in January 2021 (third round) were selected for 16 generations. The BW animals from the third round were multiplied (no selection) in Bergen op Zoom facility to reach the volumes required for the following rounds in production. Since artificial selection is a continuous and cumulative process, newer generations are expected to be superior under the same environmental conditions.

### Rearing and Data Collection

For all rounds and both lines, the experiments were carried out in a standard larval rearing cycle, which lasted 7 days. The rearing phase started with five-day-old BSF larvae dosed on rearing crates with feed. Larval density (number of larvae per crate), feed rations (grams of feed per larvae), and feed type varied across rounds as reported in Table 1. On the seventh day of the experiments (harvest day at the farm), the larvae were separated from the leftover substrate by sieving. For all rounds and both lines, average larval weight (mg) was measured at harvest day. Larvae were randomly picked from each crate from different locations within the crate. Larvae were cleaned, and 100 larvae were randomly sampled. The sample of 100 larvae was weighed on an analytical weighing scale (Nimbus NBL 4201e, Adam Equipment, UK) to determine the average larval weight using the following formula:

$$\mathrm{Average larval weight}\left(\mathrm{mg}\right)=\frac{\mathrm{sample weight}\left(\mathrm{mg}\right)}{\mathrm{number of larvae in the sample}}$$

Wet crate yield (kg), which reflects the total larval yield of the crate, was obtained after sieving out the substrate. For nutritional composition analysis, an equivalent volume of larvae from all crates per pattern was sampled, mixed, and stored in a plastic jar. The samples were first frozen at −18°C and then shipped to Nutrilab B.V. (Giessen, Netherlands). The samples were analysed for moisture (in accordance with EC-152/2009), dry matter (DM; derived from total mass and moisture), crude protein (in accordance with NEN-EN-ISO 16634), and crude fat (in accordance with EC-152/2009).

Results from the nutritional composition analysis were available for all rounds. These results, expressed as percentages, were multiplied by the average wet crate yield to obtain average crate outputs (kg) for dry matter, crude protein, and crude fat per pattern per round. These outputs, along with the feed nutritional information, were used to derive feed and protein conversion ratios.

The feed conversion ratio (FCR) was estimated on dry matter basis using the formula:

$$\mathrm{FCR}=\frac{\mathrm{input feed}\left(\mathrm{kg}\right)}{\mathrm{crate yield}\left(\mathrm{kg}\right)}$$

As data were available only for the rearing phase, input feed represents the amount of feed administered only during the rearing phase, and the crate yields are not adjusted for the young larvae weight at the beginning of the rearing phase. As a result, the absolute FCR estimates were slightly underestimated, but consistently in both lines.

The protein conversion ratio (PCR) was estimated on dry matter basis using the formula:

$$\mathrm{PCR}=\frac{\mathrm{input protein in feed}\left(\mathrm{kg}\right)}{\mathrm{protein in crate yield}\left(\mathrm{kg}\right)}$$

As data were available only for the rearing phase, input protein in feed represents the protein component in the rearing feed only. Similarly, protein in young larvae during the start of the experiment was not accounted for in this estimation. As a result, the absolute PCR estimates were slightly underestimated, but consistently in both lines.

All results are presented in percentages and represent the difference in performance between the BW and the BP lines. The following formula was used:

$$\mathrm{Performance differenc}e_{i}\left(\%\right)=\frac{BW_{i}-BP_{i}}{BP_{i}}*100$$

where i represents crate averages for larval weight, wet crate yield, DM crate yield, crude protein per crate, crude fat per crate, FCR, and PCR per pattern per round.

### Statistical Analysis

Using the data on the six experimental rounds, the average larval weight per crate was analysed fitting a linear model:

$$y_{\mathrm{ijk}}=µ+L_{i}+F_{k}+R_{j}+\varepsilon_{\mathrm{ijk}}$$

where µ represents the overall average larval weight, L is the fixed effect of the line (*i* = BW, BP), F is a covariate for the adjusted feed ration (feed amount/estimated larvae density), R is the fixed effect of the round (*j* = 1, 6), and *ε* represents the random error term. Analysis of variance (ANOVA) was used to test the relationship between average larval weight and the explanatory variables fitted in the model. In addition, the fixed effect of density was tested, yet not found significant. Indirectly however, the effect of density was included in the model as a part of adjusted feed ration (feed amount/estimated larvae density). Interactions between the variables were also tested. All interactions were found to be non-significant with one exception, where Line*Round interaction was significant in rounds three and four. However, the residuals from the model with the significant interaction did not fit the normality assumptions required for a linear model.

A paired samples t-test was used to test the difference between the two population means for measured traits across rounds (for round four, as multiple densities were tested for the BW line, only the BW pattern with the same density as the BP line were kept for the analysis, i.e., 4-BW-D5 and 4-BP-D5). All variables were tested for normality (Shapiro-Wilk test) and homoscedasticity (F-test for equality of variances) prior t-test. For data not satisfying normality or homoscedasticity, a Wilcoxon signed ranks test was used. All analyses were carried out in R statistical computing environment version 4.0.0 (R Core Team, 2020).

## Results and Discussion

The BW line performances per round are reported in Table 2 (and per variable in Supplementary Figure S1, Supplementary Material). From round one to round three, a control group from the BP line was kept under the same environmental conditions. Results from the first three rounds showed that the BW line outperformed the BP line with 28% increase in average larval weight, 24% increase in wet crate yields, 26% increase in dry matter crate yields, 20% increase in crude protein per crate, and 26% increase in crude fat per unit crate. In round one and round three, the BW line received higher feeding ration than the BP line. In round two, where the BW and the BP lines received the same feed rations, the BW line still outperformed the BP line. Therefore, this showed that the BW line outperforms the BP line at same feeding regime, yet when feeding the BW line according to its needs, it is shown that the growth potential of the BW line is higher.

**TABLE 2:** Descriptive statistics of performance differences between the Body weight line compared to the Base population line. The differences are expressed as percentage relative to the Base population line within round for feed ration (grams of feed per larva), average larval weight, wet crate yield, dry matter (DM) crate yield, crude protein per crate, crude fat per crate, feed conversion ratio (FCR) and protein conversion ratio (PCR). Larval density is coded in ascending order from D1 to D6.

Round	Feed ration (%)	Larval density	Larval weight (%)	Wet yield (%)	DM yield (%)	Crude protein (%)	Crude fat (%)	FCR	PCR
1	17	D4	22	33	29	27	34	−10%	−8%
2	0	D1	23	11	13	3	10	−11%	−3%
3	41	D2	40	29	35	30	33	4%	8%
4	53	D3	53	13	11	21	6	13%	3%
4	33	D5	37	23	16	35	11	15%	−1%
4	12	D6	18	24	14	26	8	17%	5%
5	30	D5	43	30	24	31	20	4%	−1%
6	22	D5	35	38	27	33	22	−4%	−8%

**FIGURE 2:**
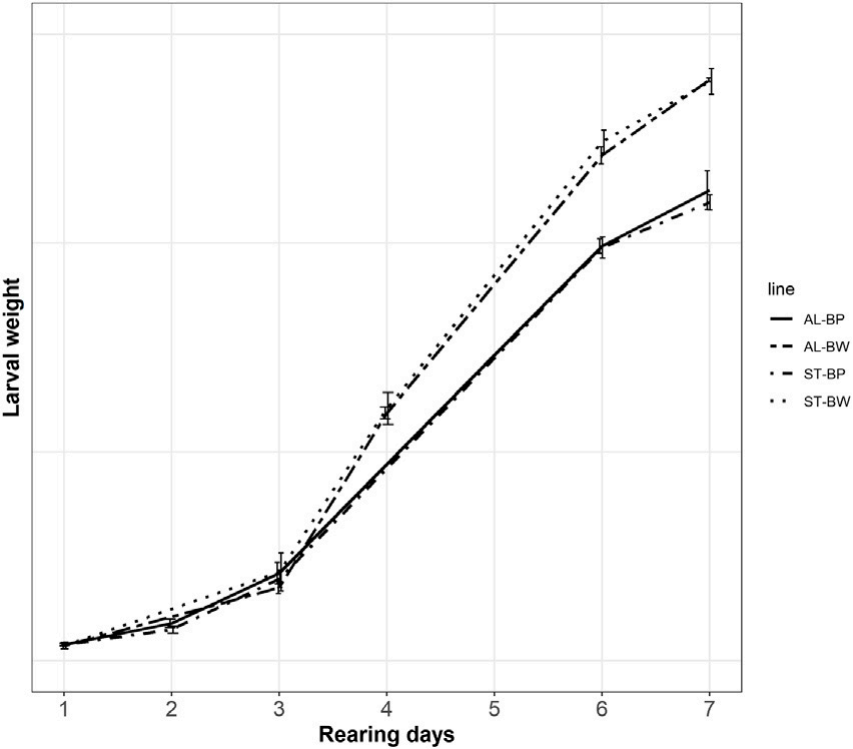
Growth curve of larvae of the BW and the BP lines over rearing days during the feeding trial (error bars indicate standard deviation). AL-BP: The Base population line at *ad-libitum* feeding ration; AL-BW: The Body weight line at *ad-libitum* feeding ration; ST-BP: The Base population line at standard feeding ration; ST-BW: The Body weight line at standard feeding ration.

The need to feed the BW line more than the BP line was further supported by results of an independent feeding trial, which was carried out separately in the Genetics lab. The results of the feeding trial are shown in Figure 2. The difference within a line between feeding treatments, as well as the difference between two lines within the same feeding treatment, was compared. Figure 2 shows the average larval weights of the BW and the BP lines measured on two different feeding treatments, the standard and the *ad-libitum* feeding rations. The figure shows how growth curves of the two feeding treatments within the lines overlap. In other words, growth curves of AL-BW (*ad-libitum* feeding, the Body weight line) and ST-BW (standard feeding, the Body weight line) overlap, as well as growth curves of AL-BP (*ad-libitum* feeding, the Base population line) and ST-BP (standard feeding, the Base population line). This indicates that both standard feeding rations for the BW (1.82 g feed/larva) and the BP (1.67 g feed/larva) groups were appropriate to the animals’ needs and comparable to *ad-libitum* feeding ration (2.5 g feed/larva). Results showed that the two lines had different growth patterns. The BP line larvae could only reach a certain weight at the end of the rearing cycle, even when fed *ad-libitum*. On the other hand, the same is true for the BW larvae but, when compared to the BP line, they eat more feed and gain more weight in the same time span. These differences in growth rate are more discernible by the end of the experiment, where both ST-BW and AL-BW larvae are ca. 25% heavier compared to ST-BP and AL-BP at harvest day (rearing day 7, Table 3).

**TABLE 3:** Average larval weight at different rearing days during the feeding trial.

Line	Rearing days			
	1	3	6	7
AL-BP	−0.7%	7.1%	0.4%	2.6%
AL-BW	−7.2%	−10.5%	22.3%	26.7%
ST-BW	−7.2%	8.6%	25.7%	26.3%

Data are expressed as deviation (%) from the Base population line at standard feeding ration (ST-BP). AL-BP: The Base population line at *ad-libitum* feeding ration; AL-BW: The Body weight line at *ad-libitum* feeding ration; ST-BW: The body weight line at standard feeding ration.

From round four onwards, the results from the BW line were compared to the parallel BP production data instead of a parallelly running control BP line. The goal from round four onwards was to optimize the BW line performances in industrial settings. Round four explored optimal larval density for the BW line reared in industrial settings. A constant increase in larval density (Table 1) resulted in a steady decrease in average larval weight (Table 2). This is most likely caused by the difference in feed availability among the density treatments, rather than the direct effect of density, i.e., with higher larval densities, less feed was available per larva. This is due to the fixed intrinsic characteristics of the production system, where the total biomass (larvae + feed) volume is fixed for a given crate size. Therefore, only the relative proportion of larvae and feed can be changed in a fixed volume. Consequently, an increase in larval density did not translate in a proportional crate yield increase. This is shown in the crate yields obtained with D6 compared to D5 in round four. While D6 had a density 18% higher than D5 (Table 1), wet crate yields were only 1% higher and dry yields were even 2% lower (Table 2). Hence, depending on the production system, an optimal balance between density, feed rations and yield should be sought. Based on results from round four, density D5 (Table 1) was selected for the subsequent rounds.

**FIGURE 3:**
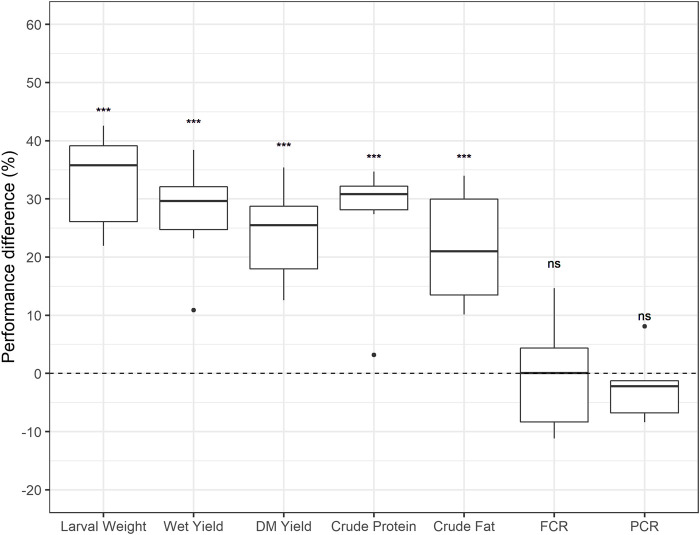
Performance differences in average crate outputs between the Body weight line and the Base population line. Boxplots represent the distribution of the results from all the six rounds for average larval weight, wet crate yield, dry matter (DM) crate yield, crude protein per crate, crude fat per crate, feed conversion ratio (FCR) and protein conversion ratio (PCR); ***: *p* < 0.001, **: *p* < 0.01, ns: *p* > 0.05. Note: *t*-test boxplots per variable available in Supplementary Figure S1.

On average, in rounds five and six in fully automated production, the BW line output over the BP line had a 39% increase in larval weight, 34% increase in wet crate yield, 26% increase in dry matter crate yield, 32% increase in crude protein per crate, and 21% increase in crude fat per crate (Table 2). For efficiency parameters, where lower values indicate better conversion, results were non-significant (Figure 3). However, results from round six were in a favourable direction, with a PCR improvement of −4% and an FCR improvement of −8%. Further improvements in conversion efficiency might require direct selection on these traits, and the optimization of crate density, feeding rations and optimal climate conditions.

Considering the results reported in last two rounds and the fact that the genetic improvement program started 2 years prior (in March 2019), we estimated the annual improvement for BSF under production settings to be between 17.5 and 21.5% for larval weight. This is considerable in terms of annual improvement in body weight compared to other species. For example: 5% increase per year was reported for broiler chicken (Hume et al., 2011), yet in a multi-trait breeding program. Similarly, in mealworms annual pupal body mass improvements was 8% per year (Morales-Ramos et al., 2019). It is however crucial to consider that the difference in the yearly improvement is dependent on the generation interval of the species, the genetic architecture of the trait and the modality of selection program (e.g., mass selection, the use of selection indexes, optimal contribution selection). The generation interval for BSF is 5–6 weeks, which is very short compared to other species like broiler (20–24 weeks), pigs (52–54 weeks), and mealworms (18–24 weeks). Since genetic gain is accumulated per generation, a shorter generation interval contributes to the higher improvement rate per year for BSF.

Results of the linear model, analyzing the average larval weight per crate, are reported in Table 4. All variables had a significant effect on average larval weight. The focus of the research is on the variance explained by genetics, represented by the variable Line. The variable Line explained the largest part (49%) of the variance in the model. This reveals the inherent genetic difference in average larval weight of the BW line compared to the BP line.

**TABLE 4:** Analysis of variance for average larval weight. Df: degrees of freedom; Sum Sq: Sum of Squares; Mean Sq: Mean sum of squares.

Variable	Df	Sum sq	Mean sq	*F* Value	Pr (>F)
Line	1	127,391	127,391	1347.21	*p* < 0.001
Adj. Feed ration	1	88,661	88,661	937.63	*p* < 0.001
Round	5	15,777	3155	33.37	*p* < 0.001
Residual	314	29,692	95	—	—

The genetic improvement program aimed to increase the body weight in black soldier fly larvae, meanwhile other traits were monitored for potential indirect selection responses. t-test results showed a significant difference between the BW and the BP mean performances for average larval weight [*t* (5) = 10.988, *p* < 0.001]. Indirect selection responses were also found in this study, with significant differences in wet crate yield [*t* (5) = 6.0535, *p* < 0.001], DM crate yield [*t* (5) = 7.4597, *p* < 0.001], crude protein per crate [*t* (5) = 5.2401, *p* < 0.01], and crude fat per crate [*t* (5) = 5.7807, *p* < 0.01] (Figure 3). Differences between the two lines were not significant for FCR (*p* = 1) and PCR [*t* (5) = −0.7734, *p* = 0.47] (Figure 3). The results showed that selecting for increased body weight did not significantly influence efficiency parameters of the BW line compared to the BP line. Various studies in livestock report FCR to have a favourable moderate negative genetic correlation with average daily gain, and a positive correlation with body weight (Schenkel et al., 2004; Case et al., 2012; Homma et al., 2021). Yet, in this study, no proof of a correlated response was found in BSF. Similarly, past studies on fish showed that selection for growth did not have significant effect on FCR (Sanchez et al., 2001; Ogata et al., 2002; Mambrini et al., 2004). This indicates that improving FCR or PCR might require direct selection on these traits.

The results showed that there was a substantial genetic improvement in larval weight in BSF, which directly translated to higher yields in crates. We successfully realized genetic improvement in the Genetics lab, which translated with minimal losses when tested in an industrial setting. With an increase in differences in environmental conditions between breeding and production, the risk of genotype by environment interaction increases. To mitigate this risk, it is important to keep the conditions as similar as possible. However, this does not mitigate the risk caused by differences between different production facilities, that might call for tailor-made selection programs for specific environments.

In addition to higher (crate) yield, other potential goals for genetic improvement would be increased fertility, reduced FCR and favorable nutritional profile. When the breeding goal is more complex, multi-trait selection can be implemented to realize balanced genetic improvement. In this study, we have clearly demonstrated that artificial selection can lead to substantial changes in larval weight and crate yields. This has economic and environmental implications. The economic implications of achieving a higher output depend on the production system. A higher yield per year results in a reduction of the fixed costs per ton of product. Fixed costs include depreciation costs for capital investments and operating costs of labor and utilities which are not related to actual output. An improvement of FCR would reduce the feed costs per ton of product. BSF are used as a waste remediation technology. A reduction in FCR implies that more insect product can be produced from the same waste stream. However, when the production facility is not capable of producing more product and it receives a tipping fee for taking waste, a lower FCR results in lower amount of waste that is converted. In that particular case, a lower FCR might not be economically attractive, but the facility would also be less dependent on input sources. Collectively, improvements in crate yield and FCR can increase the profitability in existing and new BSF facilities. Genetic improvement can help to not only improve economic performance but also to reduce GHG emissions (De Haas et al., 2021). Energy and feed are the main factors that determine the GHG emissions of BSF production (Smetana et al., 2019). Energy sources are particularly impactful for GHG if they are fossil fuels. This impact can be mitigated by using renewable energy sources, optimizing processes and genetic improvement. Since feed has a major impact on GHG emissions, lowering FCR is an important trait for a genetic improvement program from an environmental point of view. From our experiments, we learned that selection for larval weight only had a small indirect and non-significant effect on FCR. Increasing the response for FCR requires moving from a single trait approach as applied in our experiment to a multi-trait approach as commonly applied in other livestock species.

Genetics offers the potential to improve the economics and environmental footprint of BSF production. To realize this potential, investments in research are needed to answer some of the fundamental questions that lie at the basis of the implementation of a genetic improvement program which delivers sustainable progress in multiple traits. A better understanding is needed of the genetic correlations between traits and the number of genes contributing to the genetic variation. Understanding of the size and origin of genotype by environment interaction needs to be improved to determine the most appropriate environment for running the genetic improvement program and the role of breeding programs tailored to specific environments. In addition, research to unlock the potential of insect genetics is needed on reproductive biology, genetic diversity, and optimization of genetic improvement programs for insects, not just to improve response to selection, but also to limit inbreeding increase. To ensure responsible innovations, it is important to involve all stakeholders in the insect sector in setting the priorities for the research agenda. In response to the call on transparency from society, several organizations active in livestock genetics have adopted ethical guidelines and a code of best practice of animal breeding and reproduction (FAO, 1992; EFFAB, 2020). These initiatives could benefit companies that are interested in taking advantage of insect genetics.

## Conclusion

The results shown demonstrate that substantial genetic improvement in BSF can be realized through selection. We successfully realized genetic improvement in the Genetics Lab, which translated when tested in industrial settings. Opportunities for further improvements of artificial selection in BSF are presented. Realizing these improvements requires both fundamental and applied Research & Development activities. These activities will benefit from a close collaboration with industry to allow testing of concepts in an industrial setting. The implications of our findings are not restricted to BSF, but also apply to the whole insect for food and feed industry. This study shows that genetic improvement programs have a role to play in the insect farming industry, parallel to what it did in plants and in other animal species.

## Data Availability

The datasets presented in this article are not readily available because the raw data includes production information, which is confidential. Requests to access the datasets should be directed to ES, eric.schmitt@protix.eu.
