# Supplementary material for: Long-Term Artificial Selection for Increased Larval Body Weight of Hermetia illucens in Industrial Settings
Source: Front Genet. 2022 Jun 15;13:865490. doi: 10.3389/fgene.2022.865490 (PMC9240604; doi:10.3389/fgene.2022.865490)
Supplement: Supplementary file 1 [file DataSheet1.docx]

Supplementary Material

# Supplementary Figures and Tables


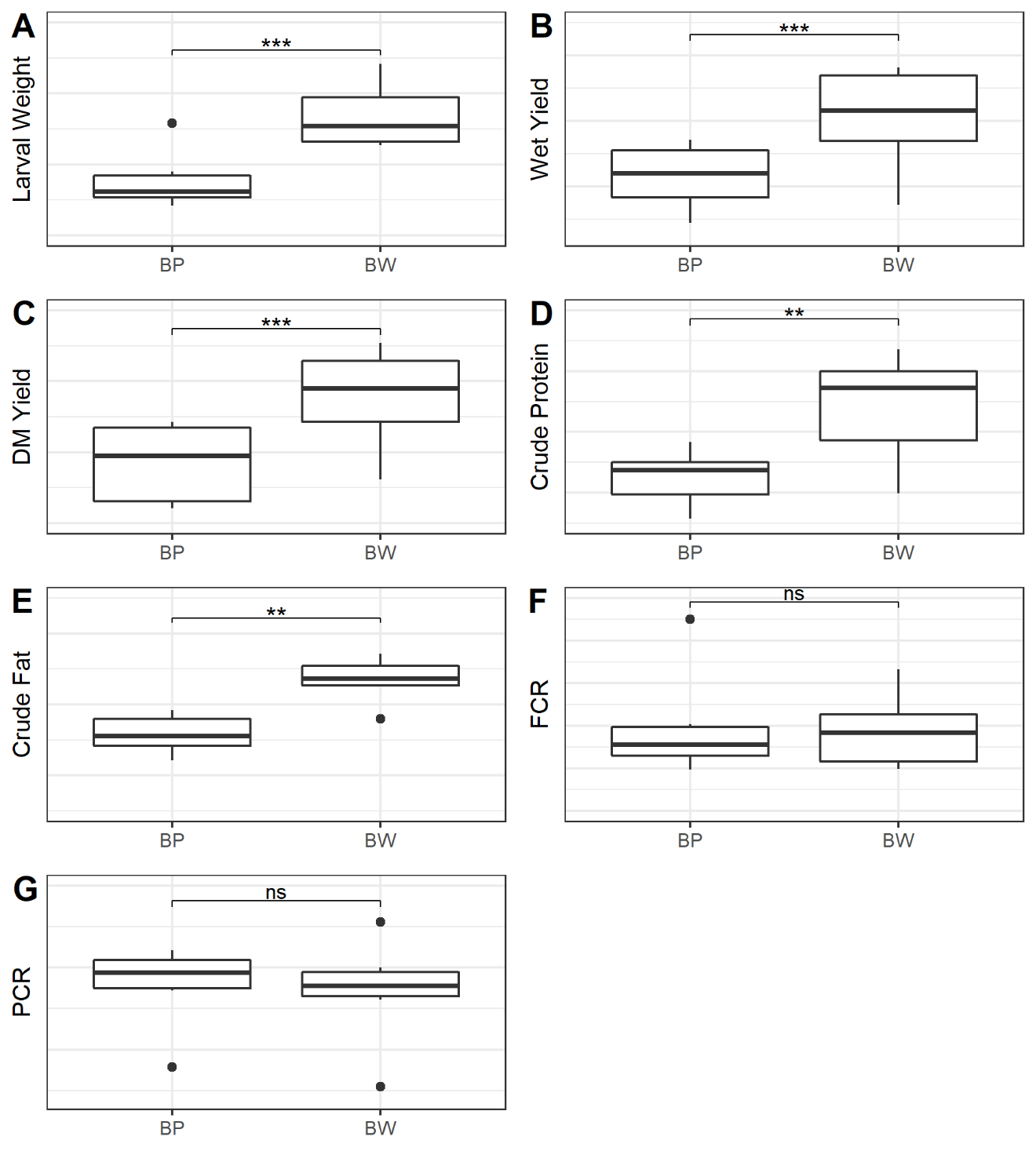


Supplementary Figure 1. Distribution of performances within phenotype across the six rounds for the body weight line (BW) and the base population line (BP). A: average larval weight; B: wet crate yield; C: dry matter (DM) crate yield; D: crude protein per crate; E: crude fat per crate; F: feed conversion ratio (FCR); G: protein conversion ratio (PCR); values on y axes are voluntarily omitted for confidentiality reasons; ***: p< 0.001, **: p< 0.01, ns: p> 0.05.
